# Women’s healthcare decision-making capacity and HIV testing in sub-Saharan Africa: a multi-country analysis of demographic and health surveys

**DOI:** 10.1186/s12889-020-09660-y

**Published:** 2020-10-22

**Authors:** Abdul-Aziz Seidu, Joseph Kojo Oduro, Bright Opoku Ahinkorah, Eugene Budu, Francis Appiah, Linus Baatiema, Edward Kwabena Ameyaw, Francis Sambah

**Affiliations:** 1 Department of Population and Health, College of Humanities and Legal Studies, University of Cape Coast, Cape Coast, Ghana; 2 College of Public Health, Medical and Veterinary Sciences, James Cook University, Townsville, Queensland Australia; 3 The Australian Centre for Public and Population Health Research, Faculty of Health, University of Technology Sydney, Sydney, NSW Australia; 4 Berekum College of Education, Berekum, BA Ghana; 5 Department of Health, Physical Education, and Recreation, University of Cape Coast, Cape Coast, Ghana

**Keywords:** Healthcare decision-making capacity, HIV, Sub-Saharan Africa, Testing, Women

## Abstract

**Background:**

Global commitment to stop Human Immunodeficiency Virus (HIV) and ensure access to HIV treatment calls for women empowerment, as these efforts play major roles in mother-to-child transmission. We examined the association between women’s healthcare decision-making capacity and uptake of HIV testing in sub-Saharan Africa.

**Methods:**

We used data from the current Demographic and Health Surveys (DHS) of 28 countries in sub-Saharan Africa, conducted between January 1, 2010 and December 31, 2018. At the descriptive level, we calculated the prevalence of HIV testing in each of the countries. This was followed by the distribution of HIV testing across the socio-demographic characteristics of women. Finally, we used binary logistic regression assess the likelihood of HIV testing uptake by women’s health care decision-making capacity and socio-demographic characteristics. The results were presented as Crude Odds Ratios (COR) and Adjusted Odds Ratios (AOR) with their corresponding 95% confidence intervals signifying precision. Statistical significance was set at *p*-value < 0.05.

**Results:**

We found that prevalence of HIV testing uptake in the 28 sub-Saharan African countries was 64.4%, with Congo DR having the least (20.2%) and the highest occurred in Rwanda (97.4%). Women who took healthcare decisions alone [COR = 3.183, CI = 2.880–3.519] or with their partners [COR = 2.577, CI = 2.335–2.844] were more likely to test for HIV, compared to those whose healthcare decisions were taken by others, and this persisted after controlling for significant covariates: [AOR = 1.507, CI = 1.321–1.720] and [AOR = 1.518, CI = 1.334–1.728] respectively.

**Conclusion:**

Sub-Saharan African countries intending to improve HIV testing need to incorporate women’s healthcare decision-making capacity strategies. These strategies can include education and counselling. This is essential because our study indicates that the capacity of women to make healthcare decisions has an association with decision to test for their HIV status.

## Background

Human Immunodeficiency Virus (HIV) and Acquired Immune Deficiency Syndrome (AIDS) constitute one of the world’s most serious public health problems [1]. Worldwide, an estimated 1.8 million newly infected HIV cases were recorded in 2017 [1]. This was made up of 180,000 children predominantly living in sub-Saharan Africa (SSA), who were infected by their HIV-positive mothers during pregnancy, childbirth, or breastfeeding [2]. HIV testing, especially among women aged 15–49, is a challenge in SSA [3, 4]. Some of the challenges identified in previous studies include but not limited to HIV-related stigma from health professionals, HIV status disclosure dilemma, unintended pregnancy, intimate partner violence, HIV and environmental structural barriers, distress, and fear related to maternal and child health [3, 5]. The few studies on this subject target women who access health services in specific countries [6].

Global commitment to stopping new HIV infections and ensuring access to treatment calls for women empowerment, due to the major role it plays in mother-to-child-transmission [1, 7]. Women’s decision-making capacity is imperative for ensuring HIV testing and addressing the pandemic [8], especially in SSA. The importance of women’s decision-making capacity in the area of HIV testing has been highlighted to build women’s confidence to prevent HIV infection, especially mother to child infection [9]. Women’s access to HIV testing depends substantially on the level to which they have been empowered to make decisions [10]. It is believed that a woman who is empowered culturally, politically, or professionally has the confidence to decide on HIV testing, as she does not depend on her husband or partner to make decision to test for HIV or not [11].

The Theory of Gender and Power (TGP) by Connell [12] could guide the exploration of association between women’s healthcare decision-making capacity and HIV testing. The TGP postulates that power dynamics between men and women are manifested in three major structures: sexual division of labour, sexual division of power, structure of social exposure and affective attachment [12, 13]. These structures provide a description of the gendered relationships between men and women which explain the power and role dynamics drifting more dominance to the males making the females subservient. Hence, a woman’s decision to go for testing may depend on whether there is equality between her and the partner, societal expectations, and norms in relation to who should decide on going for testing and whether or not there is male dominance in the home. According to the theory, although there is a distinction between these structures, they overlap and thus may not be considered in isolation from one other. These structures are also maintained through societal and institutional social mechanisms [13, 14]. These societal and institutional social mechanisms include specific socio-demographic characteristics of individuals.

For women, the specific socio-demographic characteristics which, together with healthcare decision making capacity, influence HIV testing include age, education, wealth, employment, residence, and parity [8, 15]. In SSA, literature on women’s decision-making capacity and HIV testing is limited to specific countries such as Tanzania [8], Ethiopia [16], and South Africa [17] and mostly among women who are accessing healthcare services at health facilities [18, 19]. This suggests the paucity of studies on women’s decision-making and HIV testing in SSA. It is, therefore, imperative to conduct a study in a broader context covering the entire SSA in order to empower and imbue women with a positive attitude for HIV testing. In light of the foregoing, we examined women’s healthcare decision-making capacity and HIV testing in SSA. We hypothesized that women who have the capacity to take decisions on healthcare alone or with their partners are more likely to test for their HIV status. Findings from such a multi-country study will provide evidence on the need and how to strengthen existing strategies to improve HIV testing and counselling by tackling women’s decision-making capacity.

## Methods

**Fig. 1:**
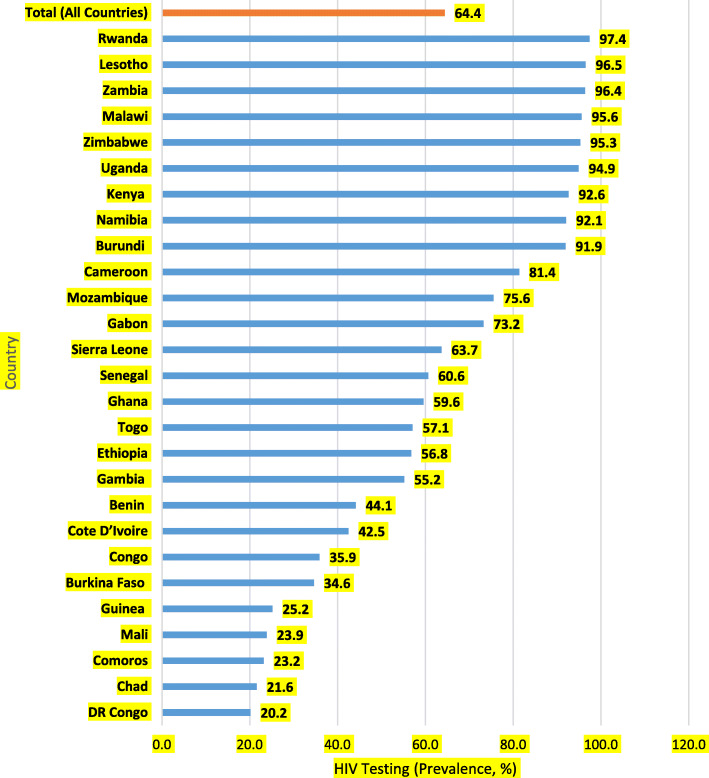
Prevalence of HIV testing among women in SSA

We used pooled data from the current Demographic and Health Surveys (DHS) conducted from January 1, 2010 and December 31, 2018 in 28 countries in SSA (see Fig. 1). DHS is a nationwide survey collected every five-year period across low- and middle-income countries. DHS focuses on maternal and child health by interviewing women of reproductive age (15–49 years) and men between 15 and 64 years. DHS surveys followed the same standard procedures – sampling, questionnaire development, and data collection. However, data cleaning, coding, and analysis were done in this study for cross-country comparison. The survey employed a stratified two stage sampling technique. The initial stage involved the selection of points or clusters (enumeration areas [EAs]), followed by a systematic sampling of households listed in each cluster or EA. For this study, the women’s file of the DHS data was used. All the participants were women in their reproductive age (15–49), who were usual members of the selected households and/or visitors who slept in the household on the night before the survey. In this study, only women in unions who had complete information on all the variables of interest were included (*N* = 195,307). We relied on the “Strengthening the Reporting of Observational Studies in Epidemiology” (STROBE) statement in writing the manuscript.

### Definition of variables

#### Outcome variable

The outcome variable was HIV testing uptake. It was derived from the question “have you ever tested for HIV?” and the responses were coded as “1=Yes and 0=No”.

#### Explanatory variables

Thirteen explanatory variables were considered in our study, including the key explanatory variable (women’s decision-making on healthcare). Women’s decision-making on healthcare was derived from the question “Who usually makes decisions about healthcare for yourself: you, your (husband/partner), you and your (husband/partner) jointly, or someone else?” The responses were categorised as respondent alone, respondent and husband/partner, husband/partner alone, someone else, and other. These were recoded into respondent/woman alone = 1, respondent and husband/partner = 2, husband/partner alone = 3 and other = 4 (family members and friends).

Besides women’s decision-making on healthcare, 12 additional variables were included in the study. These are survey country, age, educational level, marital status, religion, wealth status, place of residence, parity, occupation, and exposure to mass media (radio, television, and newspaper). Apart from survey country which was predetermined based on the geographical scope of the study, the selection of the rest of the variables was based on their association with HIV testing uptake in previous studies [6–8, 20–25]. Marriage was recoded into ‘married (1)’ and ‘cohabiting (2)’. Occupation was captured as ‘not working (0)’, ‘managerial (1)’, ‘clerical (2)’, ‘sales (3)’, ‘agricultural (4)’, ‘household/domestic (5)’, ‘services (6)’, and ‘manual (7)’. We recoded parity (birth order) as ‘zero birth’(0), ‘one birth (1)’, ‘two births (2)’, ‘three births (3)’, and four or more births (4)’. Lastly, religion was recoded as ‘Traditional religion (1)’, ‘Christianity (2)’, ‘Islam (3)’, ‘No religion (4)’, and ‘Other religion (5)’ (e.g. Hinduism, Buddhism, Atheism, Juddaism, Taoism, Confucianism, Sikhism).

### Statistical analyses

The data was analysed with STATA version 14.2 for Mac OS. The analysis was done in three steps. The first step was the computation of the prevalence of HIV testing uptake in SSA (see Fig. 1). The second step was a cross-tabulation by which we calculated the prevalence and proportions of HIV testing across the socio-demographic characteristics (see Table 1). Then, we conducted a bivariate logistic regression (Model I) and multivariable regression (Model II) analyses to assess the predictors of HIV testing among women in SSA (see Table 2). All frequency distributions were weighted and the survey command (svy) in STATA was used to adjust for the complex sampling structure of the data in the regression analyses. There was multicollinearity between knowing a place for HIV testing and HIV testing uptake. Due to this, it was taken out of the analysis. After it was taken out, there was no evidence of multicollinearity among the remaining variables (Mean VIF = 1.35, Maximum VIF = 1.70, Minimum VIF = 1.05). All results of the logistic regression analyses were presented as Crude Odds Ratios (CORs) and Adjusted Odds Ratios (AORs) at 95% confidence intervals (CIs).

**Table 1:** Socio-demographic characteristics and prevalence of HIV testing among women in SSA

Variables	Weighted	Weighted	HIV testing	
	N	%	No(%)	Yes(%)
**Age (*p*** **< 0.001)**				
15–19	11,471	5.9	45.8	54.2
20–24	33,274	17	31.5	68.5
25–29	42,429	21.7	30.5	69.5
30–34	38,061	19.5	30.5	69.5
35–39	31,407	16.1	34.8	65.2
40–44	22,159	11.4	39.6	60.4
45–49	16,506	8.5	49.4	50.6
**Education (*p*** **< 0.001)**				
No formal education	75,990	38.9	54.8	45.2
Primary	66,398	34	24.3	75.7
Secondary	45,690	23.4	21.6	78.4
Higher	7229	3.7	7.1	92.9
**Marital status (*p*** < 0.001**)**				
Married	158,959	81.4	36.1	63.9
Cohabitation	36,348	18.6	29.5	70.6
**Religion (*p*** **< 0.001)**				
Traditionalist	4110	2.1	67.3	32.8
Christianity	118,237	60.5	24.1	76.0
Islam	65,606	33.6	51.0	49.0
No religion	4063	2.1	50.9	49.1
Other religion	3291	1.7	43.3	56.7
**Wealth status (*p*** **< 0.001)**				
Poorest	36,699	18.8	46.3	53.7
Poorer	39,136	20	42.0	58.0
Middle	39,007	20	38.5	61.5
Richer	40,035	20.5	30.2	69.8
Richest	40,430	20.7	18.9	81.1
**Occupation (*p*** **< 0.001)**				
Not working	52,787	27	36.2	63.8
Managerial	8145	4.2	11.9	88.1
Clerical	1397	0.7	10.1	89.9
Sales	34,960	17.9	38.0	62.0
Agriculture	68,862	35.3	39.4	60.6
Household/domestic	3581	1.8	16.2	83.8
Services	9491	4.9	28.8	71.2
Manual	16,084	8.2	26.1	73.9
**Place of Residence (*p*** < 0.001**)**				
Urban	65,252	33.4	25.1	74.9
Rural	130,055	66.6	39.8	60.2
**Parity (*p*** **< 0.001)**				
0	11,690	6.0	47.5	52.5
1	30,413	15.6	28.5	71.5
2	33,833	17.3	27.5	72.5
3	30,030	15.4	30.4	69.6
4+	89,340	45.7	39.8	60.3
**Frequency of Reading newspaper (*p*** **< 0.001)**				
Not at all	163,600	83.8	39.0	61.1
Less than once a week	17,748	9.1	14.1	86.0
At least once a week	12,978	6.7	14.0	86.0
Almost every day	981	0.5	13.4	86.7
**Frequency of Listening radio (*p*** **< 0.001)**				
Not at all	77,374	39.6	42.3	57.7
Less than once a week	39,034	20	34.5	65.6
At least once a week	73,723	37.8	28.1	71.9
Almost every day	5176	2.7	24.2	75.8
**Frequency of Watching television (*p*** **< 0.001)**				
Not at all	117,427	60.1	39.3	60.7
Less than once a week	23,423	12	34.7	65.3
At least once a week	45,498	23.3	27.0	73.0
Almost every day	8960	4.6	18.2	81.8
**Decision maker on health (*p*** **< 0.001)**				
Respondent alone	35,943	18.4	23.5	76.5
Respondent and husband/partner	78,870	40.4	27.9	72.1
Husband alone	78,951	40.4	46.8	53.2
Other	1543	0.8	50.1	49.9

*P values are from chi-square test

*Other religion (e.g. Hinduism, Buddhism, Atheism, Juddaism, Taoism, Confucianism, Sikhism)

**Table 2:** Logistic regression analysis on women’s healthcare decision-making capacity and HIV testing in SSA

Variables	Model I COR (95%CI)	Model II AOR (95%CI)
**Decision maker on health**		
Respondent alone	3.183***[2.880–3.519]	1.507***[1.321–1.720]
Respondent and husband/partner	2.577***[2.335–2.844]	1.518***[1.334–1.728]
Husband alone	1.108*[1.005–1.223]	1.285***[1.130–1.460]
Other	Ref	Ref
**Age**		
15–19	Ref	Ref
20–24	1.813***[1.737–1.893]	1.351***[1.273–1.433]
25–29	1.876***[1.799–1.956]	1.335***[1.255–1.421]
30–34	1.901***[1.822–1.983]	1.229*** [1.150–1.312]
35–39	1.583***[1.516–1.652]	0.964 [0.900–1.032]
40–44	1.242***[1.187–1.299]	0.658*** [0.613–0.708]
45–49	0.866***[0.826–0.908]	0.383***[0.355–0.413]
**Education**		
No Education	0.061***[0.056–0.068]	0.259***[0.227–0.294]
Primary	0.224***[0.204–0.247]	0.378***[0.333–0.430]
Secondary	0.266***[0.241–0.294]	0.562***[0.496–0.637]
Higher	Ref	Ref
**Marital status**		
Married	0.772***[0.753–0.791]	0.914***[0.879–0.951]
Cohabitation	Ref	Ref
**Religion**		
Traditionalist	0.469***[0.428–0.514]	0.778***[0.688–0.879]
Christianity	3.073***[2.871–3.290]	1.070 [0.969–1.181]
Islam	0.920* [0.859–0.985]	0.787***[0.710–0.873]
No religion	0.971 [0.887–1.062]	0.771***[0.682–0.873]
Other religion	Ref	Ref
**Wealth status**		
Poorest	Ref	Ref
Poorer	1.282***[1.247–1.317]	1.244***[1.200–1.289]
Middle	1.531***[1.489–1.575]	1.380***[1.329–1.433]
Richer	2.104***[2.044–2.166]	1.719***[1.646–1.795]
Richest	4.146***[4.014–4.283]	2.561***[2.418–2.713]
**Occupation**		
Not working	0.190*** [0.158–0.227]	0.589***[0.471–0.737]
Managerial	0.796* [0.658–0.963]	0.879 [0.693–1.117]
Clerical	Ref	Ref
Sales	0.178*** [0.149–0.213]	0.739**[0.591–0.926]
Agriculture	0.160*** [0.133–0.191]	0.571*** [0.456–0.716]
Household/domestic	0.588*** [0.480–0.719]	0.634***[0.491–0.820]
Services	0.254*** [0.211–0.305]	0.734**[0.583–0.924]
Manual	0.308*** [0.257–0.370]	0.678***[0.540–0.852]
**Residence**		
Urban	Ref	Ref
Rural	0.505*** [0.494–0.515]	0.732***[0.707–0.759]
**Parity**		
0	Ref	Ref
1	2.267*** [2.169–2.370]	4.193***[3.941–4.461]
2	2.351*** [2.250–2.456]	4.382***[4.114–4.668]
3	2.056*** [1.968–2.149]	4.401***[4.120–4.702]
4+	1.345*** [1.294–1.399]	4.788*** [4.488–5.108]
**Newspaper**		
Not at all	0.241*** [0.196–0.297]	0.656***[0.510–0.843]
Less than once a week	0.974 [0.788–1.205]	0.844 [0.654–1.088]
At least once a week	0.904 [0.730–1.119]	0.767*[0.594–0.991]
Almost every day	Ref	Ref
**Radio**		
Not at all	0.468*** [0.439–0.499]	0.757***[0.693–0.828]
Less than once a week	0.661*** [0.619–0.706]	0.958 [0.874–1.051]
At least once a week	0.897*** [0.840–0.956]	0.982 [0.896–1.075]
Almost every day	Ref	Ref
**Television**		
Not at all	0.328*** [0.309–0.349]	0.631***[0.575–0.691]
Less than once a week	0.421*** [0.395–0.450]	0.766***[0.696–0.842]
At least once a week	0.606*** [0.569–0.646]	0.824*** [0.752–0.903]
Almost every day	Ref	Ref
**Survey country**		
Burkina Faso	0.019***[0.015–0.025]	0.0382***[0.0284–0.0515]
Benin	0.029***[0.022–0.038]	0.0430***[0.0319–0.0579]
Burundi	0.413***[0.310–0.551]	0.890 [0.657–1.205]
DR Congo	0.010***[0.007–0.012]	0.010***[0.007–0.013]
Congo	0.020***[0.015–0.027]	0.0208***[0.0154–0.0282]
Cote D’Ivoire	0.027***[0.020–0.036]	0.0470***[0.0348–0.0635]
Cameroon	0.159***[0.119–0.210]	0.269***[0.200–0.363]
Ethiopia	0.048***[0.036–0.063]	0.0931*** [0.0692–0.125]
Gabon	0.099***[0.075–0.132]	0.0969***[0.0713–0.132]
Ghana	0.054***[0.040–0.071]	0.0637***[0.0472–0.0860]
Gambia	0.045***[0.034–0.059]	0.0907***[0.0671–0.122]
Guinea	0.012***[0.010–0.016]	0.0225***[0.0167–0.0305]
Kenya	0.456***[0.342–0.608]	0.726*[0.536–0.983]
Comoros	0.011***[0.008–0.015]	0.0131***[0.00952–0.0179]
Lesotho	Ref	Ref
Mali	0.0114***[0.008–0.015]	0.0180***[0.0133–0.0244]
Malawi	0.788 [0.591–1.050]	1.432*[1.058–1.938]
Mozambique	0.112***[0.084–0.149]	0.269***[0.198–0.365]
Namibia	0.421***[0.310–0.570]	0.446***[0.322–0.616]
Rwanda	1.351 [0.987–1.850]	2.146***[1.543–2.983]
Sierra Leone	0.064***[0.048–0.084]	0.157***[0.117–0.212]
Senegal	0.056***[0.042–0.074]	0.130***[0.0967–0.176]
Chad	0.010***[0.008–0.013]	0.0177***[0.0130–0.0241]
Togo	0.048***[0.037–0.064]	0.0831***[0.0615–0.112]
Uganda	0.676**[0.506–0.902]	1.124 [0.828–1.525]
Zambia	0.964 [0.713–1.305]	1.561** [1.136–2.144]
Zimbabwe	0.734*[0.543–0.994]	0.810 [0.589–1.114]
N	195,307	195,307
R^2^	–	0.368

*COR* Crude Odds Ratio, *AOR* Adjusted Odds Ratio, *CI* Confidence Interval in square brackets, *Ref* Reference;

*p < 0.05, **p < 0.01, ***p < 0.001

*Other religion (Hinduism, Buddhism, Atheism, Juddaism, Taoism, Confucianism, Sikhism)

## Results

### Prevalence of HIV testing among women in sub-Saharan Africa

Figure 1 shows the prevalence of HIV testing in entirety and in each of the 28 SSA countries. Overall, the prevalence of HIV testing was 64.4%. We found that the prevalence of HIV testing ranged from 20.2% in Congo DR to 97.4% in Rwanda.

### Socio-demographic characteristics and prevalence of HIV testing

Table 1 summarises the prevalence of HIV testing across the included socio-demographic characteristics. The highest prevalence of HIV testing was among women with higher education (92.9%) and the lowest prevalence was among those who were Traditionalists (32.8%). All the socio-demographic characteristics showed statistically significant relationship with HIV testing (Table 1).

### Association between women’s healthcare decision-making and socio-demographic characteristics on HIV testing in sub-Saharan Africa 

Table 2 shows results on the association between women’s healthcare decision-making capacity and socio-demographic factors associated with HIV testing among women in SSA. The results indicate that women who took healthcare decisions alone [COR = 3.183, CI = 2.880–3.519] or with their partners [COR = 2.577, CI = 2.335–2.844] were more likely to test for HIV compared to those whose healthcare decisions were taken by others, and this persisted after controlling for significant covariates [AOR = 1.507, CI = 1.321–1.720] and [AOR = 1.518, CI = 1.334–1.728], respectively (see Model II). With the covariates, women aged 20–24 [AOR = 1.351- CI = 1.273–1.433], richest women [AOR = 2.561, CI = 2.418–2.713], those with parity 4 or more [AOR = 4.788- CI = 4.488–5.108] were more likely to test for HIV, compared to those aged 15–19, those in the poorest wealth quintile, and those with parity 0, respectively. On the other hand, women aged 45–49 [AOR = 0.383, CI = 0.355–0.413], those with no formal education [AOR = 0.259, CI = 0.227–0.294], married women [AOR = 0.914, CI = 0.879–0.951], those with no religion [AOR = 0.771, CI = 0.682–0.873], those who were not working [AOR = 0.589, CI = 0.471–0.737], those in agriculture [AOR = 0.571, CI = 0.456–0.716], those in rural areas [AOR = 0.732, CI = 0.707–0.759], those not exposed to newspaper [AOR = 0.656CI = 0.510–0.843], those not exposed to radio [AOR = 0.757,CI = 0.693–0.828], those not exposed to television [AOR = 0.631, CI = 0.575–0.691], and those in Congo DR [AOR = 0.010,CI = 0.007–0.013] were less likely to test for HIV, compared with those aged 15–19, those with higher education, those cohabiting, those belonging to other religious groups, those in clerical jobs, those in urban areas, those who read newspaper almost every day, those who listened to radio almost every day, those who watched television almost every day, and those in Lesotho.

## Discussion

This study examined the association between women’s healthcare decision-making capacity and HIV testing uptake in SSA, using the most recent DHS of 28 countries. This study was imperative since HIV testing has been noted as a challenge for some women in SSA [3] whilst some evidence suggests that decision-making capacity plays a significant role in whether a woman will test for HIV or not [11, 26]. We found that women who had high capacity to make decisions relating to their health either alone or with their partners were more likely to test for HIV. This may not necessarily imply that women who did not have the capacity do not prioritise HIV testing; instead, it may indicate their inability to translate their thoughts into action. This finding reinforces evidence from Tanzania and Nigeria that empowered women who are capable of making decisions have higher chances of HIV testing [8, 25]. In line with TGP, the finding indicates that HIV testing among women can occur in empowered women through a reduction in gender inequality, positive societal expectation of women’s decision-making capacity in marriage, and absence of male dominance. Our finding suggests that efforts to halt HIV and vertical transmission need to target decision-making capacity of women in the household and community level. Additionally, facility-based interventions such as supplying and subsidising the cost of antiretroviral vaccines will be necessary. Active community engagement geared towards women empowerment especially in the areas of decision-making, therefore, needs to be prioritised. A recent systematic review indicated that encouragement from peers could enhance ability of women to undergo HIV test [27] and this is a key community-level strategy. Testing for HIV was high among women from Rwanda. This is not surprising in light of the upsurge in health sector reforms in Rwanda in the past few years [28]. Another factor that might have accounted for the high likelihood of HIV testing in Rwanda may be Rwanda’s continual efforts marked by 3.0% stabilised HIV prevalence among the general population and 50% reduction in new HIV infection rate [29]. Rwanda is more likely to further improve in HIV testing and reduction with the introduction of self-HIV-testing kit which can be purchased over the counter [29]. There is the need to encourage women in DR Congo to utilise HTC.

In relation to the covariates, the study revealed that HIV testing declines as women advance in age and parity but increases among women with higher educational attainment, those with richest wealth quintile, urban women, women who have knowledge on HIV, and those who are exposed to media. The findings on age and parity are consistent with earlier studies conducted in a number of SSA countries. For instance, one study from Namibia reported that the likelihood of HIV testing generally declined as women advanced in age [22]. It is possible that women who are advanced in age and possibly with high parity will feel that they have limited exposure to HIV due to possible decline in sexual intercourse [30]. Such women may feel less motivated to test for HIV. In terms of socio-economic status, education, urban living, and wealth status have been identified as strong predictors for HIV testing in previous studies [21, 23, 25, 31–37]. Education and wealth status are forms of empowerment. Hence, it is not surprising that such women exercise their decision-making capacity in a way that can improve their health status, which is testing for HIV. This indicates that policies on mother-to-child-transmission (MTCT) and plans to halt HIV need to consider measures that can enhance economic standing of women. This is in line with the Health Belief Model, which argues that gaining consciousness about a health condition is the first step to inform the needed precaution [37, 38]. Women of high socio-economic status (education and wealth) are also more likely to have knowledge of HIV. This finding is consistent with previous evidence from Burkina Faso and Cambodia that knowledge about HIV have greater implication on whether women will undergo the test or otherwise [23, 39, 40]. Such knowledge can be obtained from exposure to media, which can enhance HIV testing as indicated in previous studies [38–40, 41, 42].

### Strengths and limitations

This study offers a true account of women’s decision-making capacity on healthcare and HIV testing emerging from most recent national surveys of 28 countries in SSA. The large sample and rigour of the methodological and analytical approaches are significant strengths of the study. However, the study is not devoid of limitations. The predictors exposed in this study only account for less than half of the variability in HIV testing in SSA and that other factors on the demand and supply side are likely to explain the bigger proportion of the variability in HIV testing across the region. Again, women were only asked if they had tested for HIV without any validation and as a result recall bias could occur. However, these do not outweigh the rigour of the study in light of the acknowledged strengths. Previous studies have established strong associations between knowing a place to get tested, comprehensive knowledge of HIV/AIDS, and discriminatory attitude towards people living with HIV and HIV testing, however, these variables were not consistent across the countries we included in our analysis. Therefore, we did not add them to our study. Knowing a place to get tested showed high level of multicollinearity and was dropped.

## Conclusions and policy implications

The study has demonstrated that ensuring women’s healthcare decision-making capacity has the propensity to increase HIV testing uptake among women in SSA. SSA countries that seek to improve HIV testing need to incorporate women’s healthcare decision-making strategies into the available policies because our study indicates that as more women are able to make decisions in their household relating to their health, their chances of HIV testing increases. In addition to focusing on provision of care (i.e., providing HIV test kits, targeting household and community level structures), prioritising women’s decision-making capacities can contribute positively to HIV testing. Much of this effort is required in DR Congo as women from that country had the least likelihood of HIV testing. Other category of women to target when developing measures to increase HIV testing are the poorest and married women, women over 45 years, those not having formal education, agricultural workers, rural women, nulliparous women, women having more than four births, and women having limited contact with mass media (television, radio, and newspaper). Various context-specific mass media channels can, therefore, be used to reach the identified category of women depending on available resources.

## Data Availability

The dataset is freely available at https://dhsprogram.com/data/available-datasets.cfm.

## Abbreviations

AIDS: Acquired Immune Deficiency Syndrome
AOR: Adjusted Odds Ratio
CI: Confidence Interval
COR: Crude Odds Ratio
DHS: Demographic and Health Surveys
EA: Enumeration Areas
HIV: Human Immunodeficiency Virus
SSA: Sub-Saharan Africa
TGP: Theory of Gender and Power
